# Toxicity of Nanoparticles in Biomedical Application: Nanotoxicology

**DOI:** 10.1155/2021/9954443

**Published:** 2021-07-30

**Authors:** Chukwuebuka Egbuna, Vijaykumar K. Parmar, Jaison Jeevanandam, Shahira M. Ezzat, Kingsley C. Patrick-Iwuanyanwu, Charles Oluwaseun Adetunji, Johra Khan, Eugene N. Onyeike, Chukwuemelie Zedech Uche, Muhammad Akram, Mervat S. Ibrahim, Nihal M. El Mahdy, Chinaza Godswill Awuchi, Kaliyaperumal Saravanan, Habibu Tijjani, Uchenna Estella Odoh, Mohammed Messaoudi, Jonathan C. Ifemeje, Michael C. Olisah, Nebechi Jane Ezeofor, Chukwudi Jude Chikwendu, Chinwe Gloria Ibeabuchi

**Affiliations:** ^1^Africa Centre of Excellence in Public Health and Toxicological Research (ACE-PUTOR), Nutritional Biochemistry and Toxicology Unit, University of Port-Harcourt, Port Harcourt, Rivers State, Nigeria; ^2^Department of Biochemistry, Faculty of Science, University of Port Harcourt, East-West Road, P.M.B 5323, Port Harcourt, Rivers State, Nigeria; ^3^Department of Biochemistry, Faculty of Natural Sciences, Chukwuemeka Odumegwu Ojukwu University, Uli, Anambra State 431124, Nigeria; ^4^Department of Pharmaceutical Sciences, Sardar Patel University, Vallabh Vidyanagar - 388 120, Gujarat, India; ^5^CQM–Centro de Química da Madeira, MMRG, Universidade da Madeira, Campus da Penteada, 9020-105 Funchal, Portugal; ^6^Department of Pharmacognosy, Faculty of Pharmacy, Cairo University, Kasr El Ainy Street, 11562 Cairo, Egypt; ^7^Department of Pharmacognosy, Faculty of Pharmacy, October University for Modern Sciences and Arts (MSA), 6th of October 12566, Egypt; ^8^Applied Microbiology, Biotechnology and Nanotechnology Laboratory, Department of Microbiology, Edo University Iyamho, PMB 04 Auchi, Edo State, Nigeria; ^9^Department of Medical Laboratory Sciences, College of Applied Medical Sciences, Majmaah University, Majmaah 11952, Saudi Arabia; ^10^Health and Basic Sciences Research Center, Majmaah University, Majmaah 11952, Saudi Arabia; ^11^Department of Medical Biochemistry and Molecular Biology, Faculty of Basic Medical Sciences, University of Nigeria, Enugu Campus, Nsukka, Nigeria; ^12^Department of Eastern Medicines, Government College University, Faisalabad, Pakistan; ^13^Department of Pharmaceutics and Industrial Pharmacy, Faculty of Pharmacy, October University for Modern Sciences and Arts (MSA), 6th of October 12566, Egypt; ^14^School of Natural and Applied Sciences, Kampala International University, Kampala, Uganda; ^15^PG and Research Department of Zoology, Nehru Memorial College (Autonomous), Puthanampatti–621 007, Bharathidasan University, Tiruchirappalli, Tamil Nadu, India; ^16^Department of Biochemistry, Natural Product Research Laboratory, Bauchi State University, Gadau, Nigeria; ^17^Department of Pharmacognosy and Environmental Medicines, Faculty of Pharmaceutical Sciences, University of Nigeria, Nsukka, Nigeria; ^18^Nuclear Research Centre of Birine, Ain Oussera, P.O. Box 180, Djelfa 17200, Algeria; ^19^Chemistry Department, University of Hamma Lakhdar, B.P. 789, El-Oued 39000, Algeria; ^20^Department of Medical Biochemistry, Faculty of Basic Medical Sciences, Chukwuemeka Odumegwu Ojukwu University, Uli, Anambra State 431124, Nigeria; ^21^Department of Food Technology, School of Applied Science and Technology, Federal Polytechnic, Oko, Anambra State, Nigeria

## Abstract

Nanoparticles are of great importance in development and research because of their application in industries and biomedicine. The development of nanoparticles requires proper knowledge of their fabrication, interaction, release, distribution, target, compatibility, and functions. This review presents a comprehensive update on nanoparticles' toxic effects, the factors underlying their toxicity, and the mechanisms by which toxicity is induced. Recent studies have found that nanoparticles may cause serious health effects when exposed to the body through ingestion, inhalation, and skin contact without caution. The extent to which toxicity is induced depends on some properties, including the nature and size of the nanoparticle, the surface area, shape, aspect ratio, surface coating, crystallinity, dissolution, and agglomeration. In all, the general mechanisms by which it causes toxicity lie on its capability to initiate the formation of reactive species, cytotoxicity, genotoxicity, and neurotoxicity, among others.

## 1. Introduction

Nanotoxicology is an aspect of nanoscience that deals with the study of the adverse effects of engineered nanomaterials or nanoparticles on living organisms. The ever-increasing application of engineered nanoparticles for biomedical application has raised serious concerns about their safety in humans. Nanoparticles (NPs) are widely used as nanomedicine and nanocarriers of drugs, due to their small size and exclusive properties [1, 2]. However, their size [3], morphology, surface functional groups [4], and dose-dependent properties [5] may also be responsible for their toxicity towards normal, healthy human cells, tissues, and organs. Several studies have shown that chemically synthesized NPs have high toxicity on human cells due to the presence of synthetic chemicals as surface functional and capping agents, compared to biosynthesized nanoparticles that possess biocompatible surface functional groups [6]. On the contrary, certain biosynthesized nanoparticles also exhibit toxicity upon reaction with cells, while disintegrating into its simpler forms or due to accumulation [7, 8]. The scope of nanotoxicology is aimed at identifying potential hazards that are useful for the safety evaluation of nanomedicines. This review consulted current literature and presented recent information about the toxic effects of nanoparticles.

## 2. Properties of Nanoparticles That Influence Toxicities

The NPs properties that influence toxicity are size, surface area, shape, aspect ratio, surface coating, crystallinity, dissolution, and agglomeration.

### 2.1. Size and Surface Area

As nanoparticle size decreases, the ratio of surface area to volume exponentially increases, which in turn increases biological and chemical reactivities [9]. For instance, when the size of the NP decreased from 30 to 3 nm, the number of surface molecules expressed increased from 10 to 50% [10]. The cytotoxicity of nanomaterials results from the interaction between the nanomaterial surface and cellular components. Thus, even when nanoparticles have the same chemical composition, they can have a significantly different level of cytotoxicity depending on surface area and particle size. In other words, NPs have higher toxicity in comparison to the bigger particles with similar compositions.

Chao et al. [11] reported silver NPs' size-dependent acute toxicity in BALB/c mice after intraperitoneal administration of silver nanoparticles, which had diameters of 10, 60, and 100 nm. Histopathological changes such as the thymus cortex apoptosis; focal necrosis, single-cell necrosis, vacuolation, and congestion in the liver; and congestion in the spleen were only seen after administering 10 nm silver nanoparticles and not for 60 and 100 nm silver nanoparticles. Thus, smaller nanoparticles have greater acute toxicities in mice.

Du et al. [12] investigated cardiovascular toxicity of different sizes of amorphous silica NPs (90, 60, and 30 nm) and 600 nm of fine silica NPs after intratracheal instillation in rats. The silica concentrations in serum and heart were evaluated using inductively coupled plasma optical emission spectroscopy. Blood levels of inflammation-related proteins, cytokines, and tumor necrosis factor were found higher in rats administered fine silica particles.

In the case of the administration of nanoparticles via inhalation, different sizes of nanoparticles showed specific distribution patterns in the respiratory tract. The toxicological evaluation of the response to inhaled nanoparticles requires knowledge of the dose of nanomaterials deposited in the respiratory tract. Braakhuis et al. [13] showed size-dependent pulmonary inflammation after inhalation of 15 and 410 nm of silver NPs. The NPs inhaled were not removed sufficiently compared to the large particles through the mechanisms of macrophage clearance in lungs and that could cause lung damage.

Lopez-Chaves et al. [14] evaluated subcellular location, toxic effects, and tissue distribution of three different gold NPs' sizes. They used particles of 10, 30, and 60 nm sizes and assessed *in vivo* distribution after intraperitoneal administration in the rat. The gold nanoparticles of 10 and 30 nm crossed the membrane of the nucleus, consequently favoring breaks in DNA. These 10 and 30 nm gold NPs seemingly accumulate more in liver, kidney, and intestine than 60 nm gold nanoparticles. The highest accumulation of 60 nm particle was observed in the spleen. Thus, the larger the sizes of gold NP, the higher they accumulate in the spleen.

Nanoparticles' absorption from the GI tract is a multistep process; a function of mucus layer interaction, enterocytes contact and assimilate through a cellular entry or paracellular transport [15]. NPs less than 100 nm get absorbed by intestinal cells, unlike the bigger NPs (300 nm) [16]. Smaller NPs' (100 nm) absorption in lymphatic tissues is higher than in cells of the intestine [16]. Although some studies stated otherwise, endocytosis remains the main mechanism mostly common for NPs' uptake into epithelial cells of the intestine [16].

In summary, NPs have larger surface areas and higher particle numbers per unit mass in comparison with the bigger particles (Table 1). The body reacts in a different way with similar mass composed of billions of NPs in comparison with many microparticles. The engineered nanoparticles possess high surface reactivity, as well as high surface area, which could result in producing higher reactive oxygen species level, thus leading to cytotoxicity and genotoxicity [17, 18].

### 2.2. Shape

**Figure 1:**
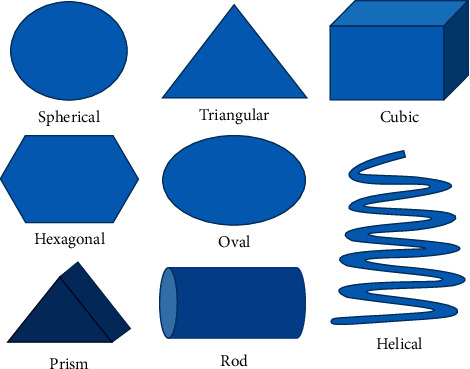
Typical shapes of nanomaterials.

Shape is an important factor of nanoparticles that play a vital role in determining their biological reactivity as well as toxicity. The typical shapes of nanoparticles are sphere, cylinder, cube, sheet, or rod (Figure 1). The shape of the nanoparticle is important in determining its cellular uptake.

The cellular uptake of carbon nanomaterial of spherical shape and tubes of multi-graphitic sheets was observed in epithelial tissues of both gut and gill, but not of cube-shaped carbon nanoparticles [19]. Silver nanoplates were found to be more harmful than silver nanospheres in zebrafish (*Danio rerio*) embryos [20]. The spherical nanoparticles are taken up in greater numbers in cells compared to the other shapes [21]. Gold nanorods cause less accumulation of autophagosome than gold nanospheres [22]. Steckiewicz et al. [23] examined the cytotoxic properties of gold NP of stars, rods, as well as spheres against human fetal osteoblast, osteosarcoma, and pancreatic duct cell line using MTT assay. The star-shaped gold nanoparticles are the most cytotoxic against human cells. Both cytotoxicity and anticancer potentials of gold nanoparticles depend on shape. Mesoporous silica nanoparticles have shown potential as a drug carrier in oral drug delivery. The needle-shaped nanoparticles exhibit more toxicity than those with spherical shape, because of their improved multiple endocytic mechanisms, internalization rates, and more efficient adhesiveness to the surface of the target cell [24–26].

### 2.3. Aspect Ratio

A nanoparticle aspect ratio is the width to height ratio. An aspect ratio of 1 represents a spherical particle, while nanotube has an aspect ratio close to zero. The greater the NPs' aspect ratio, the higher the toxicity of the NPs [27]. Aspect-ratio-dependent toxicity is generally observed in the lung. The nanofibers with about 150 nm thickness and 2, 5, and 10 *µ*m length show asbestosis, mesothelioma, and lung cancer, respectively [27]. Muller et al. [28] studied the pulmonary toxicities of carbon nanotube with a high aspect ratio in Sprague-Dawley rats following administration directly into the trachea. Carbon nanotube samples caused significant protein exudation and granulomas on the peritoneal side of the diaphragm [28].

Li et al. [29] systematically studied the effect of NPs of mesoporous silica with different aspect ratios of 5, 1.75, and 1 on their *in vivo* toxicity, excretion, and biodistribution after administration through the oral route. With a reduction in the aspect ratio, systematic absorption through organs, e.g., the small intestine, increased while the excretion via urine reduced. Renal toxicity which depends on shape of silica nanoparticles was reported [29].

### 2.4. Crystallinity

The type of crystalline structure may affect the toxicity of nanomaterials. Polymorphs, the different crystalline structures of the same chemical composition showed different chemical and physical properties. Lai et al. [30] reported cytotoxicity of 10-hydroxycamptothecin (HCPT) nanoparticle dispersions, which depends on the polymorph, in both *in vivo* and *in vitro* studies. Three 10-hydroxycamptothecin polymorphic nanoparticle dispersions, i.e., pan-cake, prismatic, and needle forms, were made and characterized. The cytotoxicity results indicated that all the different HCPT nanoparticles' cellular toxicities depended on size and shape. However, the needle-shaped HCPT nanoparticles are more potent in apoptotic response in cancer cells despite similar cellular uptakes as prismatic nanoparticles. This effect may explain the preference for polymorph with different thermodynamic properties, including lattice energy. Andersson et al. [31] also reported titanium dioxide NPs' uptake and toxicity in A549 lung epithelial cells, which were polymorph-dependent. These reports lay emphasis on the significance of the accurate characterization of the polymorphic form (crystalline structure) of nanoparticles for reliable assessment of toxicity.

### 2.5. Surface Coating or Surface Functionalization

Surface coatings of nanoparticles are applied in order to modify its properties. The surface of a particle (the “core”) is covered with a variety of layer(s) (the “shell”). The objective of the surface coating may be to tailor its stability, wettability, dissolution, or functionality. The surface coating can convert noxious particles to be nontoxic while less harmful particles may become more toxic due to bioavailability. Xu et al. [32] performed an *in vitro* evaluation of the toxicity of iron oxide nanoparticles coated with silica (Fe_3_O_4_/SiO_2_ NP) on the cells of HeLa and A549. Reports indicate that surface passivation of NPs decreases the alteration of iron homeostasis and oxidative stress. As a result of this, there is overall toxicity reduction during cell internalization when compared to nanoparticles that are not passivated.

In a separate study, the iron oxide NPs coated with polyethyleneimine (PEI) were reported to significantly exhibit higher uptake than PEGylated iron oxide nanoparticles in both cancer cells and macrophages which resulted in severe cytotoxicity [33]. PEI-coated iron oxide NPs were more efficiently internalized than PEGylated iron oxide NPs despite having the same nanoparticle sizes, which could be the result of the cationic NPs' affinity to the protein domains or negative head groups of phospholipids on cellular membranes. Consequently, it is imperative to put surface coatings of NPs into consideration in toxicity studies.

### 2.6. Dissolution

The dissolution ability of nanoparticles is a significant property that determines safety, uptakes, and associated toxic mechanism. Two identical NPs of similar composition and size may have completely different behavior in dissolution, depending on different surface modification [34]. Nanoparticles that undertake media dissolution before uptake by the organisms usually have clear ion channels and ion transporters as the preferred cellular entry route.

### 2.7. Agglomeration

Nanomaterials are likely to agglomerate in solution due to their high free surface energy [35]. To avoid agglomeration, nanomaterials are shielded with protective agents. The toxicity of nanomaterials is also dependent on whether or not agglomeration occurred. The agglomeration of nanoparticles could be a potential inducer of inflammatory lung conditions in humans [36]. The agglomeration-dependent toxicity of nanomaterials is more commonly observed in carbon nanotubes and oxide nanoparticles. The well-dispersed carbon nanotubes have been reported as having less toxicity than the agglomerated carbon nanotubes [37]. Zook et al. [38] showed the significance of agglomeration control by demonstrating that large silver NPs agglomerate significantly causing less hemolytic toxicity compared to small agglomerates.

## 3. General Mechanism of Nanoparticle Toxicity

The general mechanism by which metallic oxide nanoparticle induces toxicity is a joint function of the properties of the nanoparticle and its corresponding ability to induce ROS, and cause toxicity to cells, genes, and neurons.

### 3.1. Nanoparticle-Induced Oxidative Stress

Oxidative stress is among the commonly reported stresses that nanoparticles induce following exposure on a cellular level. Oxidative stress can be broadly defined as a lack of balance between antioxidants' activities and the production of oxidants [39]. A state of oxidative stress arises via increase in ROS production favored over antioxidants [40]. ROS are generally produced in the form of by-products of biochemical reactions, including neutrophil-mediated phagocytosis, enzymatic metabolism of cytochrome P450, and mitochondrial respiration [41], and commonly include peroxynitrite (ONOO−), nitric oxide (NO), hydroxyl radical (∙OH), hydrogen peroxide (H_2_O_2_), and superoxide radical (O_2_∙−) [41]. The ROS attack nucleic acids, proteins, lipids, and most vital biomolecules which can lead to an NADPH-like system activation, electron transport chain impairment, mitochondrial membrane depolarization, and damage to the mitochondrial structure [42].

Oxidative stress constitutes significant adverse effects in the use of nanoparticles, as it may generate oxidants and have the capacity to stimulate ROS generation partly as a result of relative stability of free radical intermediates, which occur on particles' reactive surfaces, or as NPs-induced cellular response, or redox-active groups caused by NPs functionalization, especially with the ability of the NP to interfere with cellular uptake [43]. Such imbalance induced by nanoparticles directly or indirectly can lead to drastic effects that may lead to cytotoxicity [44]. ROS induced by NPs can cause damage to genetic materials, including cross-linking of DNA, breakage of DNA strand, and genetic mutations. NPs can also increase ROS production by activating inflammatory cells, including neutrophils [45].

Zinc Oxide NPs (ZnONPs) are widely applied for various purposes ranging from fillers, a component of creams, powders, dental creams, absorber of UV radiation, and biosensors [46]. Nevertheless, studies have shown that zinc oxide can result in oxidative stress leading to damage on a cellular level. A study was conducted on human liver cells (HepG2) that are exposed to 14–20 lg per ml of ZnONP and are found to cause oxidative stress-mediated damage to DNA and ROS-triggered mitochondria-mediated apoptosis HepG2 [47]. ZnONPs also reduce cell viability and trigger apoptosis in primary astrocytes along with increased levels of intracellular ROS [48]. According to Hou et al. [49], ZnONPs induce the failure of minichromosome maintenance with a corresponding DNA replication disorder in different periods (G1, M, and G2 phase) in the cell cycle pathway.

Super paramagnetic iron oxide nanoparticles, which have various uses in magnetic resonance imaging distinction, improvement, immunoassays as well as drug targeting systems for cancer, were found to cause oxidative stress in addition to disturbance in iron homeostasis exposures of the core of the iron oxide and can cause oxidative stress that could be linked with disorders in neurological system [50–52].

Silver NP (AgNp) was found in several consumer items owing to their excellent anti-microbial activities. However, several reports have concluded that they have cytotoxic properties owing to oxidative stress. One report attributed oxidative stress-mediated programmed cell death of AgNPs in *Candida albicans* through the accumulation of intracellular ROS as well as other targets of the cells leading to changed ultrastructure, cellular morphology, ergosterol content, membrane microenvironment, and membrane fluidity [53]. Another study conducted on mice administered treatment orally using AgNps coated with polyvinylpyrrolidone (PVP-AgNPs) and reported permanent alterations in genes and DNA damage in several tissues [54]. Finally, the silver species oxidation in AgNPs after their quick releasing from decreased silver-rich NP after being autophaghed by lysosymes results in cellular toxicity modulated by ROS generation [55]. The same holds true for gold nanoparticles (AuNPs) which are widely used in cancer treatments; however, they have been reported to have oxidative stress-induced cytotoxicity on several cell lines, including the cells of HeLa, HepG2, and PMBC by generating ROS [56, 57].

Ahamed et al. [58] carried a study and concluded that Bi_2_O_3_ nano particles cause dose-dependent apoptosis and cytotoxicity in cells of MCF-7. However, they found that supplementing external antioxidants, N-acetyl-cysteine, altered the Bi_2_O_3_ nanoparticles' cytotoxicity effectively, thereby suggesting that Bi_2_O_3_ nano particles caused the cytotoxicity by alteration of redox homeostasis.

In addition to metallic NPs, nonmetallic nanoparticles can also cause oxidative stress. Ceramic NPs which are usually applied in drug delivery were shown to induce oxidative stress leading to cytotoxicity in the brain, heart, liver, and lungs, and also carcinogenic and teratogenic properties [59]. Also, silica nanoparticles (SiNP) were shown to initiate a time- and dose-dependent NO/NOS imbalance and oxidative stress, resulting in inflammation and dysfunction of endothelium [60]. Carbon nanotubes (CNT) were also shown to induce oxidative stress-related toxicity. Shvedova et al. [61] demonstrated that in addition to free radicals, CNT cellular uptake induced oxidation of polyunsaturated fatty acids and caused cellular apoptosis.

### 3.2. Cytotoxicity of Nanoparticles: Biochemical and Molecular Mechanisms of Cytotoxicity

In addition to cytotoxicity induced by ROS generation discussed earlier, cytotoxicity induced by nanoparticles can be caused by various physicochemical, biochemical, and molecular mechanisms.

#### 3.2.1. Physicochemical Mechanisms

As noted earlier, particle size could contribute to cytotoxic potency because smaller nanoparticles typically possess larger surface areas which enable interactions with components of the cells, including carbohydrates, fatty acids, proteins, and nucleic acids. Moreover, these very small nanoparticles have more likelihood of entering cells, resulting in damage to cells [62]. Jiang et al. [62] concluded that crystal type has a significant effect on cytotoxicity with amorphous TiO_2_ being the most cytotoxic form. Particle shape was also found to have a direct effect on cytotoxicity. Rod-shaped Fe_2_O_3_ NPs produce higher responses to cytotoxicity than Fe_2_O_3_ NPs with sphere shape in a cell line of murine macrophage (RAW 264.7), along with higher levels of necrosis, ROS production, inflammatory response, and leakage of lactate dehydrogenase (LDH) [63]. In addition, CeO_2_ nanoparticles with rod shape were reported to give substantial tumor necrosis factor-alpha (TNF) and LDH release in the cell lines of murine macrophage, whereas none of the cubic or octahedron shape could give significant responses [64].

Surface charge of particles could also have an effect on the nanoparticles' cellular uptake and their interaction with biomolecules and organelles, thereby directly influencing NP cytotoxicity with the toxicity increasing as surface charge increased. A study on the cell line of human hepatoma (BEL-7402) on several iron NPs with different surface charges concluded that the more positive charge NP has more electrostatic interaction with cells leading to more endocytic uptakes [65]. This correlated with another study which concluded that positively charged ZnONPs produced more cytotoxicity in cells of A549 than particles with negative charges despite having similar size and shape [66], which was attributed to the interaction of particles of positive charges with the Glycosaminoglycan molecule (which is negatively charged) in the mammalian cell membrane leading to the NP being more internalized [67], and the same scenario can be applied in positive charge NPs that interact with negative charge DNA, resulting in damage to DNA.

Dendrimers, which are widely used commercially in drug, gene, and siRNA delivery, with the anionic or PEGylated dendrimers showing low toxicity compared to cationic dendrimers [68], decrease cell integrity and permeability, and interact with the lipid bilayer of the biological membrane [69, 70]. PAMAM dendrimers have also been shown to have a cytotoxic effect because their surface amino groups impart a cationic charge, which upon endocytosis results in DNA damage, mitochondrial damage, oxidative stress, and consequently apoptosis [71].

#### 3.2.2. Molecular and Biochemical Mechanisms

The perturbation of Ca^2+^ (intracellular calcium) induced by nanoparticles is a major cause of cytotoxicity induced by NPs and linked to energetic imbalance, metabolic imbalance, and cellular dysfunctions [72]. Although Ca^2+^ is among the major signaling molecules involved in the transduction of cell signal in the regulation of cellular metabolism and energy output, its increase has a direct toxic effect on cellular mitochondria which respond in an apoptic pathway via selectively releasing cytochrome c or by improved ROS production and making an inner pore of mitochondrial membrane open, all of which lead to cell death [73]. ZnONPs increase Ca^2+^ and influx of extracellular calcium inflicted by membrane disruption through lipid peroxidation, malondialdehyde (MDA) causing cytotoxicity, and disruption in hemostasis [74]. A study by Lai et al. [75] reported a reduction in mitochondrial membrane potential (MMP) following ZnO exposure in alveolar adenocarcinoma cells (A549) and bronchial epithelial cells (BEAS-2B) of humans, indicating a higher risk of early apoptosis [75]. Li et al. [76] reported that ZnONP appeared to physically squeeze mitochondrial bodies in HaCa cell lines [76]. TiO_2_ leads to loss of mitochondrial membrane potential in lung A549 cells and neuronal cells (PC12) [77, 78]. Fe_3_O_4_ induced loss of mitochondrial membrane potential in human hepatoma cells (BEL-7402) and “human mesenchymal stem cells” (hMSCs) [65, 79]. Bi_2_O_3_ NPs were also reported to cause low MMP together with a greater bax/bcl-2 genes expression ratio, inducing cell apoptosis via the pathway of mitochondria [58]. Recently done studies showed that binding of proteins to metal oxide nanoparticles such as FeO, SiO_2_, TiO_2_, or ZnO NPs could lead to protein denaturation or minor changes in conformation, with irreversible proteins binding to NPs [80]. Also, Cu and Zn ions were shown to inactivate some metalloproteins through the dislodging of metallic ions in them [81].

#### 3.2.3. Cells Cycle Arrest

Cell divisions comprise two successive progressions, Mitosis (M), which is the nuclear division and interphase process, including G1, G2, and S phases. DNA replication takes place in the S phase and is preceded by the G1 phase within which the cells prepare for the synthesis of DNA; then it is followed by the G2 phase in which cells prepare for M. Cells within G1 phase may enter a state of resting known as G0, which is responsible for most part of the nonproliferating and nongrowing cells in humans [82].

Recently, it has been shown that nanoparticles' cytotoxic effect may not only lead to cell death but also to cell proliferation suppression that occurs once cells are arrested in at least one phase of the cell cycle (G2/M phase, S phase, or G0/G1 phase) [72]. Cells arrested within cell cycle either accumulate much damage leading to apoptosis or fix the damage [72].

Cell cycle arrest can be specific to certain types of cells at specific phases, for instance, nickel oxide NP (NiONP) exposure led to a significant decrease in G0/G1 in the cell line of A549 and a significant increase in the G0/G1 phase in the cell line of BEAS-2B. Similarly, the G2/M phase in the cell line A549 significantly increased and the G2/M in the cell line of BEAS-2B significantly decreased, whereas only the cell line of BEAS-2B significantly affected the S phase [83]. The type of nanoparticle also affects the cell cycle. In HaCa T cells, exposure to CuO and ZnO NPs led to G2/M phase arrest, whereas TiO_2_ exposure led to S phase arrest [76, 84, 85]. Fe_3_O_4_ and Al_2_O_3_ resulted in an increase in the phase of sub-G0 of hMSFs. Also, cells of A549 was arrested in the G2/M phase following exposures to ZnO, NiO, and CuO, but upon Fe_2_O_3_ exposure experienced no cell cycle change [75, 86].

### 3.3. Genotoxicity of Nanoparticles

The mechanism behind nanoparticle-associated genotoxicity is majorly due to the overproduction of reactive nitrogen (RNS) species and ROS, which results in increased oxidative stress and hence oxidative damage to the genetic material [87]. The NPs-mediated production of ROS and RNS can be due to intrinsic production, interaction with cell target, and/or inflammatory reaction. The resultant damage to the genetic material can be direct or indirect primary clastogenic or secondary (aneugenic, and DNA adduct production) genotoxicity [88]. The primary toxicity occurs due to the interaction of the NPs themselves with the DNA, whereas in the secondary genotoxicity, the genetic damage occurs as a result of ROS/RNS produced/carried by the NPs [89]. In the indirect primary clastrogenic mechanism, exocyclic DNA adducts are produced via unsaturated aldehydes produced as a result of ROS-mediated primary lipid oxidation. The secondary aneugenic mechanism's major consequence is chromosomal loss due to nondisjunctioning in the anaphase as a result of ROS and or RNS-induced protein oxidative lesions that affect the function of the mitotic apparatus [88]. Many scientific studies are in support of nanoparticles-induced genotoxicity. For example, Kisin et al. [90] reported that single-wall carbon nanotubes (SWCNTs) caused single- and double-strand DNA lesions in Chinese hamster fibroblasts (V79 cell line) at 96 *µ*g/cm^2^. In a different study, AgNPs at different concentrations were found to cause significant DNA damage in *S. cerevisiae* [91], larvae of the mulberry silkworm [92], adults of the microcrustacean *C. cornuta* [93], and abnormalities in micronuclei and nuclear of zebrafish (*D. rerio*) [94]. AgNPs' genotoxicity was also reported in microbes [95, 96] and plants [97–99]. A summary of reported *in vitro* and *in vivo* studies for the genotoxic effects of NPs is presented in Table 2.

### 3.4. Neurotoxicity of Nanoparticles

Neurotoxicity is a reversible or irreversible side effect that may affect the structure, function, or chemistry of the neurons in the nervous system [133]. Though the research community has centered its efforts on developing a brain-targeted drug delivery system using smart NPs, there is less information available on the neurotoxicity of these particles. Various research papers suggested that the neurotoxicity of the NPs is due to oxidative stress triggered by free radical activity [134, 135]. A summary of the neurotoxic effects of selected nanoparticles is presented in Table 3.

## 4. Studies on Specific Nanoparticles and Their Associated Toxicity

The basic mechanism of metal oxide nanoparticle toxicity is based on its colloidal dispersion, homeostasis alteration, and accumulation. The colloidal dispersion of metal oxide nanoparticles leads to ions of metals and oxygen, where the metals accumulate, alter the homeostasis of cells, and bind with cell organelles to cause toxic effects. The oxygen ion, being singlet oxygen, produces ROS, increases oxidative stress, and leads to lipid peroxidation. In the second case, the slow release of metal ions from the nanoparticles alters the cellular homeostasis as most of the fabricated metal oxide nanoparticles are trace and essential metals that are required for the development of cells. These alterations also cause the level of metals to increase more than their threshold limit, similar to the toxicity caused by heavy metal accumulation. In the third case, highly stable metal oxide nanoparticles accumulate either on the surface of the cells or internalize into the cells. These nanoparticles accumulate and agglomerate, which causes either 1^st^ or 2^nd^ case of toxicity or just the accumulation of high metal oxide concentration which may also lead to toxic effects.

**Figure 2:**
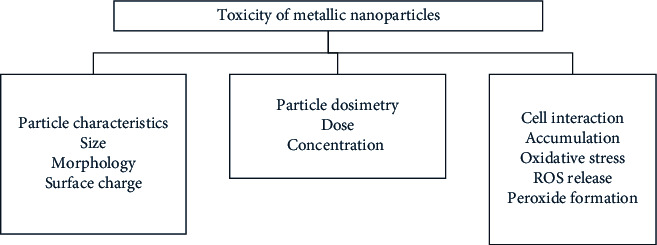
Characteristics and mechanisms of metal nanoparticles toxicity.

Metal nanoparticles exhibited cytotoxicity via three significant pathways [154] as shown in Figure 2:

1. Particle characterization
2. Dosimetry
3. Interaction with the cells

The particle characteristics are highly dependent on the synthetic approach, which can alter their size, morphology, and surface functional groups [155]. The dosimetry, including the dose and concentration of nanoparticles to initiate toxic reactions in cells, is also dependent on the physicochemical characteristics of the metal nanoparticles [155]. The cell interaction is an independent characteristic which leads to toxicity. Sometimes, the size and morphology influence the interaction of metal oxide with the cells, yet, most of the time, it is their surface charge, stability, and surface functional groups that determine the nanoparticles' cellular interaction [156]. As suggested earlier, the smaller size of these nanoparticles allows easy by-pass through the cell membrane and then to the nucleus, which may cause alterations in DNA and mitochondrial pathways, leading to serious genotoxicity. Low stable metal nanoparticles, which are dependent on the surface charge, will release free metal ions that will accumulate inside the cell organelles and inhibit their growth via oxidative stress, similar to heavy metal accumulation [157]. However, biomolecules as surface functional groups, especially phytochemicals with antioxidant properties, rather than synthetic stabilizing and a capping agent from chemically synthesized nanoparticles, helps in reducing the release of ROS from the metal nanoparticles [158], which forms peroxides and inhibits cell growth and causes cell death [159].

### 4.1. Gold Nanoparticles

Gold nanoparticles are arguably the first nanoparticles that are used in commercial materials and approved by the United States Food and Drug Administration (USFDA) as nanomedicine and nanocarrier [160, 161]. Moreover, these nanostructures possess unique size-dependent surface plasmon resonance properties that made them utilizable in biosensor applications [160]. In spite of these applications, gold nanoparticles are also considered to be toxic based on the administered dose and concentration via accumulation in cells, similar to heavy metals [159]. In recent times, several studies reported different factors that can lead to cytotoxicity towards human cells. Senut et al. [162] explored the size-dependent toxicity of gold nanoparticles towards human embryonic stem cells and their neural derivatives. Particle sizes such as 1.5, 4, and 14 nm of gold nanoparticles were used to evaluate its neuronal differentiation, viability, DNA methylation, and pluripotency. The result of the study revealed that the chemically synthesized gold nanoparticles of size below 20 nm are highly toxic to stem cells by altering cellular DNA methylation and the hydromethylation pattern [162]. Biosynthesized gold nanoparticles have shown to be an efficient anticancer drug carrier to deliver doxorubicin at the target site and cure cancer cells [163]. Thus, it is evident from these two studies that the toxicity of gold nanoparticles depends on the synthesis approach, which affects their size, morphology, topology, and surface functional group.

Recently, Jo et al. [164] evaluated the *in vitro* and *in vivo* toxicity, as well as estimated the oral absorption and tissue distribution biokinetics of orally administered, chemically synthesized gold nanoparticles using human and rat intestinal cells for 14 days. The result revealed that the gold nanoparticles were nontoxic for 24 h in terms of membrane damage, oxidative stress, and cell proliferation inhibition. However, they also revealed that these nanosized gold particles are toxic after 14 days exhibiting long-term and high concentration exposure dependent toxic reactions [164]. Similarly, there are several studies which demonstrate that gold nanoparticles are toxic to cancer cells and not to normal healthy cells [165, 166]. Semmler-Behnke et al. [167] showed that gold nanoparticles can accumulate in the fetus of a rat via maternal blood and can lead to toxicity towards the fetus [167].

### 4.2. Silver Nanoparticles

Silver nanoparticles are an important set of nanosized particles that are widely synthesized for various applications, next to gold [168]. Bulk silver possess antimicrobial activities to a certain extent, whereas nanosized silver particles possess exclusive antimicrobial properties to inhibit the growth of specific microbes, including bacteria, fungi, algae, and viruses [169]. The production of reactive oxygen species (ROS) and elevating the oxidative stress in the microbial cells are significant factors that lead to their enhanced antimicrobial activities [170]. Also, silver nanoparticles are toxic towards cancer cells by releasing ROS, specific to cancer cells [171]. The in-depth analysis of literature has revealed that only biosynthesized silver nanoparticles exhibit anticancer activity [172]. This evidence confirms that silver nanoparticles activate or enhance the immobilization of biomolecules into the cancer cells, which improve their anticancer property [173]. Even though they show toxic reactions towards microbes and cancer cells, several studies demonstrated that silver nanoparticles are less toxic or nontoxic towards healthy human cells. Kim et al. [174] examined the cytotoxicity of silver nanoparticles towards human hepatoma cells via oxidative stress. The results revealed that there is a negligible amount of free silver ions that are exposed from the nanoparticles to the cell culture. It is noteworthy that the nanoparticles are stable in the cell culture medium and agglomerate in the cellular cytoplasm and nuclei, which induces intracellular oxidative stress. In addition, the cytotoxicity of silver nanoparticles is found to be similar to the silver ions. However, the nanoparticle-mediated oxidative stress and DNA damage can be reduced by the antioxidant *N*-acetylcysteine [175]. Similarly, Ahamed et al. [176] demonstrated that the silver nanoparticles are toxic to the cells of the skin, brain, liver, lung, and reproductive and vascular systems of mammals. They also confirmed that the toxic reactions are dependent on the short- and long-term exposure of nanoparticles with the cells [111, 177].

de Lima et al. [178] stated that silver nanoparticles possess the ability to trigger inflammatory reactions in human cells. Moreover, these nanosized silver particles had the ability to cross the cell membrane and reach the nucleus which causes increasing damage to the genetic material and hence genotoxicity. They also revealed the presence of biomolecules as functional groups in biosynthesized silver nanoparticles, which reduces ROS production with their antioxidant properties. Thus, they concluded that the biosynthesized silver nanoparticles are less toxic towards normal, healthy human cells, compared to chemically synthesized nanoparticles [178]. Gaillet and Rouanet [179] examined the toxicity of silver nanoparticles after their exposure towards humans via the oral route. They revealed that the silver nanoparticles cause toxic side effects mainly in the intestinal tract and liver via oral exposure. It is noteworthy that the silver nanoparticles produce free radicals and induces oxidative damage via cellular oxidative stress, which leads to inflammatory reaction-triggered toxicity and death by apoptosis or necrosis [179]. In addition, Kennedy et al. [180] showed that the human serum albumin helps in stabilizing aqueous silver nanoparticles, which inhibits the cellular uptake of particles, thereby reducing their potential exposure towards cells to reduce toxicity [180]. Likewise, Smith et al. [181] evaluated the kinetics of silver ions and particles using RAW 264.7 macrophages, wild-type C57BL/6J mice derived bone marrow macrophages, and scavenger receptor A–deficient (SR-A^(-/-)^) mice via *in vitro* analysis. Furthermore, the accumulation of silver at an increased concentration in cells may lead to Parkinson's disease [182], silver-Russell syndrome [183], and Alzheimer's diseases [184].

### 4.3. Copper Oxide Nanoparticles

Copper oxides belong to the family of metal oxides that possess enormous applications as bulk or microparticles. When these copper oxides are reduced to the nano-size, they behave differently than their bulk counterparts and exhibit exclusive properties [185]. Copper oxide nanoparticles are widely utilized as antimicrobial agents [186] and are also explored to possess anticancer activity [187], bioimaging [188], and nano-medicinal properties (Abdelhamid). Numerous studies have revealed that the copper oxide nanoparticles are highly toxic towards microbes such as bacteria [189], fungi [190], algae [191], and viruses [185] as well as cancer cells. In spite of these exclusive biomedical properties, several reports showed that copper oxide nanoparticles are also highly toxic to normal and healthy human cells [192]. Karlsson et al. [193] evaluated the toxicity of metal oxide nanoparticles such as oxides of titanium, iron, zinc, and copper with carbon nanoparticles and multi-walled carbon nanotubes (MWCNTs) using the human A549 lung epithelial cell line. The result showed that the copper oxide nanoparticles are highly toxic to lung cells by causing oxidative lesions and damaging DNA, compared to other nanosized metal oxides, carbon nanoparticles, and MWCNTs [193]. Similarly, Studer et al. [194] compared the toxicity of stabilized copper metal and degradable copper oxide nanoparticles using Chinese hamster oocytes and HeLa cells. The results revealed that the copper oxide nanoparticles disintegrated into copper ions, and free metallic copper ions inhibit healthy cells via trojan horse-type mechanism and its physicochemical parameters, especially intracellular solubility, plays a dominating effect on their cytotoxicity [194]. In addition, Fahmy and Cormier [192] demonstrated that copper oxide nanoparticles exhibit cytotoxicity in airway epithelial cells by inducing oxidative stress. Furthermore, Alarifi et al. [195] showed that copper oxide nanoparticles are cytotoxic and genotoxic towards human skin keratinocyte cells [195]. It is noteworthy that the copper oxide nanoparticles also showed toxic reactions towards human lung epithelial cells [176], cardiac microvascular endothelial cells [196], HepG2 cells, and human skin organ culture [197]. Moreover, Atha et al. [198] demonstrated that copper oxide nanoparticles are toxic to terrestrial plant models such as *Raphanus sativus*, *Lolium perenne*, and *Lolium rigidum* by damaging their DNA [198].

In recent times, Wongrakpanich et al. [199] stated that copper oxide nanoparticles exhibit high toxicity towards lung epithelial cells, which depends on their size. Four and 24 nm sized particles were used for the study and the result demonstrated that the 24 nm sized oxide nanoparticles of copper were highly toxic to cells, compared to 4 nm sized ones. They proposed that the post-internalization events of larger nanoparticles such as disintegration into their ionic state and releasing reactive oxygen lead to their higher toxicity than smaller nanoparticles [199]. In addition, Akhtar et al. [200] showed that copper oxide nanoparticles induce dose-dependent genotoxicity by stimulating ROS generation in human lung epithelial cells [200]. Likewise, Srikanth et al. [201] showed that the copper oxide nanoparticles exhibited cytotoxicity towards Chinook salmon cells by altering their morphology and inducing oxidative stress [201]. Moreover, Ude et al. [202] evaluated the toxicity of copper oxide nanoparticles and bulk copper sulphate towards differentiated and undifferentiated Caco-2 intestinal epithelial cells. The result showed that both nano and bulk particles exhibited a concentration-dependent cytotoxicity towards undifferentiated cells. Furthermore, the study revealed that the nanoparticles stimulated the production of interleukin-8 in Caco-2 cells, decreasing the integrity of the cell barrier which helps in the translocation of copper ions [202]. In addition to cytotoxicity and genotoxicity, it is noteworthy that the copper oxide nanoparticles also induce neurotoxicity and hepatoxicity [203]. Bulcke and Dringen [204] examined the toxicity of copper oxide nanoparticles towards astrocytes in the brain and revealed that the nanoparticles rapidly undergo endocytosis-mediated accumulation in astrocytes, which increases cellular copper content, ROS production, reduces cell viability, and causes diseases due to metabolic disturbances in brain copper balance [205]. All these studies stand as evidence for the enhanced cytotoxicity of copper oxide nanoparticles and emphasize that size, morphology, dose, and concentration are the significant factors that lead to cytotoxicity. Furthermore, the mechanism of cytotoxicity involves the disintegration into its ionic states; metal ion accumulates in the cells, leading to oxidative stress and lipid peroxidation. In certain cases, the copper ions act as heavy metals [205] and exhibit trojan horse–like mechanism and bind with cell organelles including genetic material and inhibit cell development.

### 4.4. Zinc Oxide Nanoparticles

Zinc oxide nanoparticles are the most common nanosized metal oxides that are extensively utilized as anticancer agents [206]. They also exhibit enhanced antimicrobial activity, similar to copper oxide nanoparticles [207]. The microbial inhibition ability of zinc oxide nanoparticles is used to fabricate antimicrobial food packages [208], textiles [209], cotton fabric [210], and paints [211]. Moreover, the exclusive anticancer activity of zinc oxide nanoparticles enables them to be useful as nanomedicine [212], nanocarrier of cancer drugs, and optical imaging materials [213]. The zinc oxide nanoparticles also exhibited toxic reactions towards normal, healthy cells, similar to other nanosized metal oxides. However, they are less toxic compared to other metal oxide nanoparticles [214]. Kao et al. [215] evaluated the toxicity of zinc oxide nanoparticles in broncho-alveolar lavage and white blood cells. The result shows that the nanoparticles interfere with the homeostasis of zinc ions present in the body fluid. The disintegration of zinc oxide nanoparticles led to an increase in the zinc ions which eventually causes dysfunction of mitochondria, activation of caspase and apoptosis of cells [215]. Similarly, Sharma et al. [47] examined the *in vitro* cytotoxicity of zinc oxide nanoparticles towards human HepG2 liver cells. The result demonstrated that the nanoparticles exhibited apoptotic and genotoxic mediated toxicity towards liver cells. They proved that the genotoxicity is due to the damages in DNA and apoptotic toxicity is due to the ROS triggered mitochondrial damage [47]. Furthermore, Heng et al. [216] evaluated the cytotoxicity of spherical and sheet-shaped zinc oxide nanoparticles towards RAW-264.7 mouse cells, BEAS-2B human cells, and primary bone marrow–derived dendritic mouse culture cells. Both the shapes of zinc oxide nanoparticles increased the release of ROS, upregulated the expression of CD80, CD86, and released pro-inflammatory cytokines such as IL-6 and TNF-*α* which inhibits the growth of cells [216]. Likewise, Valdiglesias et al. [217] also proved that zinc oxide nanoparticles induce cyto- and genotoxicity in neurons which they proved by using SHSY5Y human neuronal cells. They emphasized that the nanoparticle did not enter into the cells and toxicity was due to the presence of nanoparticles in the medium, which lead to cell cycle alterations, apoptosis, micronuclei production, H2AX phosphorylation, and DNA damage mediated cyto- and genotoxicity. Furthermore, they added that the toxicity is dose- and time-dependent, whereas free zinc ions from the nanoparticles are not responsible for cytotoxicity in neuronal cells [217]. Several studies also reported the cytotoxicity of zinc oxide nanoparticles towards rat retinal ganglion cells [218], human epidermal cells [219], human nasal mucosa cells [220], murine macrophages [221], and human bronchial epithelial cells [222].

Recently, Ng et al. [223] evaluated the toxicity of zinc oxide nanoparticles using MRC5 human lung fibroblasts as an *in vitro* model and *Drosophila melanogaster* (fruit fly) as the *in vivo* model. The *in vitro* studies' result showed that zinc oxide nanoparticles triggered the extracellular secretion of lactate dehydrogenase, which indicates cellular damage and decreased lung cell viability. Furthermore, the presence of DNA damage-inducible transcript (DDIT3) and endoplasmic reticulum to nucleus signaling 1 (ERN1) genes revealed the generation of ROS in the lung cells after nanosized zinc oxide exposure. The *in vivo* studies also showed that the nanosized zinc oxides are lethal to *D. melanogaster* by releasing ROS, which was indicated by the presence of nuclear factor E2-related factor 2 (Nrf2) [223]. Likewise, Pati et al. [224] reported that the zinc oxide nanoparticles exhibited genotoxic, cytotoxic, clastogenic, and actin depolymerization effects by inducing ROS-mediated oxidative stress responses towards macrophages of mice. In addition, they examined their histopathological effects on adult mice, which revealed that these nanoparticles are highly toxic and lead to severe inflammation and damage to the liver, lungs, and kidneys [224]. Moreover, Abdelmonem et al. [225] synthesized zinc oxide nanoparticles with the same sizes and different surface charges by coating amphiphilic polymers such as poly (isobutylene-*alt*-maleic anhydride)-graft-dodecyl, mercaptoundecanoic acid, and L-arginine. They examined their *in vitro* cytotoxicity and uptake of these nanoparticles towards 3T3 fibroblasts and HeLa cells and the result showed that the positively charged arginine-capped nanoparticles facilitate agglomeration internalization into the cells. Thus, they proved that the surface charge of zinc oxide nanoparticles is highly significant in determining their toxicity towards normal cells [225].

### 4.5. Iron Oxide Nanoparticles

Iron oxide nanoparticles are unique metal oxides that possess magnetic as well as biomedical properties along with their enhanced biocompatibility, bioavailability, and bioactivity [226]. The superparamagnetic property of iron oxide nanoparticles makes them an essential material in the magnetically targeted delivery system for the treatment of cancer [227]. They also possess enhanced antimicrobial properties against bacteria, fungi, algae, and viruses, similar to other metal oxides; however, the mechanism of action is different from other nanosized metal oxides [228–231]. Several pieces of literature also reported that the combination of silver and iron oxide nanoparticles as nanocomposites possess exclusive and unique antimicrobial activities [232]. Even though iron oxide nanoparticles are used in biomedical applications, numerous reports showed that they also exhibit toxicity towards normal healthy cells. Singh et al. [233] reported that the superparamagnetic iron oxide nanoparticles (SPIONs) exhibited cytotoxicity via subtle cellular perturbation such as actin cytoskeleton modulation, gene expression profile alteration, iron homeostasis disturbance, impaired alterations in signaling pathways, cell regulation, DNA damage, and oxidative stress [233]. Similarly, different magnetic nanoparticles such as dextran-coated Endorem, carboxydextran-coated Resovist, lipid-coated magnetoliposomes, and citrate-coated iron oxide particles are evaluated to test their cytotoxicity against C17.2 neural progenitor cells, PC12 rat pheochromocytoma cells, and human blood outgrowth endothelial cells. The results revealed that only lipid-coated magnetoliposomes can internalize at high concentration into all the cell lines which can exhibit enhanced magnetic resonance imaging (MRI) properties [234]. Likewise, Petri-Fink et al. [235] examined the cytotoxicity of SPIONs coated with polyvinyl alcohol (PVA), vinyl alcohol/vinyl amine copolymer (A-PVA), and polyethyleneimine (PEI) towards HeLa cells. The result revealed that A-PVA coated nanoparticles showed good cell viability, compared to others. The study also shows that cytotoxicity is dependent on the colloidal stability in cell media and cellular uptake of the magnetic nanoparticles [235].

In recent times, Valdiglesias et al. [236] reviewed literature and reported the effects of iron oxide nanoparticles on healthy, normal human cells. The article emphasized that the magnetic nanoparticles are highly cytotoxic, genotoxic, developmental toxic, and neurotoxic among humans during short-term exposure and further studies are required to evaluate their long-term exposure effects [236]. In addition, Magdolenova et al. [237] evaluated the effects of surface coatings over iron oxide nanoparticles such as oleate using human lymphoblastoid TK6 cells and primary human blood cells. The result revealed that the surface-coated iron oxide nanoparticles altered their behavior and cellular uptake, and helped them to exhibit dose-dependent cytotoxicity and genotoxicity via DNA damage [237]. Similarly, Valdiglesias et al. [142] examined the toxic mechanism of iron oxide nanoparticles by analyzing several works of literature and reported that factors such as surface coating, dose, size, exposure time, and type of cells are significant to induce cytotoxicity. Furthermore, they conveyed via *in vivo* studies that these magnetic nanoparticles possess the ability to get distributed to different organs and tissues, especially cross the blood-brain barrier in the brain, and lead to acute toxicity, immunotoxicity, reproductive toxicity, genotoxicity, and neurotoxicity [142].

### 4.6. Aluminium Oxide Nanoparticles

Aluminum oxide or alumina nanoparticles are commonly used in biosensors [238], and certain biomedical applications. They are also used as antimicrobial agents, for bioimaging [239], and as anticancer nanomedicine. There are several reports available which showcased the toxicity of alumina nanoparticles towards various microbes [240] and cancer cells [241]. However, there are some literatures that report alumina nanoparticles as toxic to normal human cells. The trend in the research to evaluate the cytotoxicity of alumina nanoparticles has been increasing only in recent times. Pakrashi et al. [242] revealed the cytotoxicity of alumina nanoparticles towards freshwater algal isolate at a low concentration of exposure. Radziun et al. [243] assessed the cytotoxicity of alumina nanoparticles towards L929 murine fibroblasts and normal BJ human skin fibroblasts cells, which revealed that the alumina nanoparticles are nontoxic to these mammalian cells. Similarly, Yoon et al. [244] investigated the cytotoxicity of alumina nanoparticles for concentrations of 25–200 *µ*g/ml and an incubation time of 0–72 h using THP-1 floating cells and adherent cells such as A549, 293, and J774A.1. The results emphasized that cytotoxicity depends on the dose, time of exposure, agglomeration, sedimentation, and enhanced cellular uptake [244]. Likewise, Lin et al. [245] evaluated the cytotoxicity of 13 and 22 nm sized alumina nanoparticles using cultured human bronchoalveolar A549 carcinoma-derived cells and revealed that they are highly toxic than titanium dioxide and less toxic than cerium oxide nanoparticles via alteration in the cell membrane potential, surface chemistry, and exposure duration. In addition, Kim et al. [246] demonstrated that the alumina nanoparticles induce genotoxicity towards BEAS-2B mammalian cell lines.

Recently, More et al. [247] investigated the cytotoxicity of surface-engineered mesoporous alumina nanoparticles synthesized by sol-gel method using cetyl trimethyl ammonium bromide (CTAB). The result showed that the nanoparticles are highly stable up to 24 h and are less toxic towards Chinese Hamster Ovary (CHO) cells [247]. The reduced toxicity may be due to the enhanced protein adsorption ability [241], especially human plasma proteins [248]. Moreover, Rajiv et al. [249] evaluated the cytotoxicity and genotoxicity of cobalt, iron, silicon, and aluminum oxide nanoparticles towards human lymphocyte cell lines. The result revealed dose-dependent toxicity of all these metal oxides, where alumina nanoparticles exhibited the least oxidative stress–mediated DNA damage, compared to other metal oxide nanoparticles [249]. Another study by Asztemborska [250] evaluated and confirmed the toxicity of alumina nanoparticles towards plants via environmental transformation and bioaccumulation. In addition, it was reported that the low-dimensional alumina nanoparticles are highly toxic towards L 929 mouse fibroblast and Neuro-2a Mus musculus brain neuroblastoma cell lines via ROS production and oxidative stress.

## 5. Conclusion

Nanoparticles have found wide biomedical application due to their physicochemical and behavioral uniqueness, although concerns over their toxic effects in the biological system are now drawing the attention of the global health community. This necessitates the importance of the study and the understanding of the effects based on the cellular and molecular mechanisms by which they cause these effects. Some identified toxic mechanisms are through the induction of ROS, cytotoxicity to cells, and genotoxic and neurotoxic effects. This toxic effect depends on the type of nanoparticles, the size, surface area, shape, aspect ratio, surface coating, crystallinity, dissolution, and agglomeration. For instance, it was reported that smaller nanoparticles tend to have greater acute toxicities in animal models. It was also found that the shape or crystallinity of a nanoparticle could influence its toxicity. At this point, it is important that the toxic effects of nanoparticles be considered when synthesizing nanoparticles. Their size, shape, and other key features should be varied in order to ascertain those that work best without causing adverse effects. The consideration of the toxic effects of nanoparticles will open a new page for the synthesis of safer and more effective nanoparticles.

## Figures and Tables

**Table 1:** Particle size, surface area, and number.

Diameter of particle (nm)	Surface area of particle (*µ*m^2^ cm^−3^)	Number of particles (N cm^−3^)
5	12000	153000000
20	3016	2400000
250	240	1200
5000	12	0.15

**Table 2:** *In vitro* and *in vivo* studies for the genotoxic effect of NPs.

S/no.	Nanoparticles	*In vitro* studies	*In vivo* studies
1.	*Carbon-based NPs*		
	Single-wall carbon nanotubes (SWCNTs)	Single- and double-strand DNA lesions in Chinese hamster fibroblasts (V*79* cell line) for 3 h at 96 *µ*g/cm^2^ [90].	Genotoxicity via inhalation exposure in mice (C57BL). Causes immediate inflammatory reaction, fibrosis, oxidative stress, and hyperplasia [100].
	Multiwall carbon nanotubes (MWCNTs)	Induce apoptosis in the stem cells of mouse embryo via P53 protein activation; cause DNA damage [101–103].	Absence of genotoxicity in rats [104, 105]. Causes DNA damage in the lung cells, bone marrow, and leucocytes of mice [106, 107].

2.	*Silver NPs*: gained wide application in both food and medical purposes owing to its antimicrobial activity [108]	The micronucleus assay and comet assay confirmed the mutagenic and genotoxic effect of AgNPs [17, 109–112].	Causes DNS damage in mice lung cells and testis in size-dependent toxicity [113].

3.	*Gold NPs*: useful in gene and drug delivery as well as deep tissue imaging	AuNPs on various cell lines showed chromosomal aberration, micronuclei formation, oxidative DNA damage, and strand lesions [114–117].	Negative genotoxic results in mice but chronic and acute intraperitoneal administration of 10 and 30 nm Au NPs induced DNA damage evaluated by comet assay in the liver, blood, and cerebral cortex cells of rats [118].

4.	*Titanium dioxide (TiO*_*2*_*) NPs*: titanium dioxide (TiO_2_) NPs porous TiO_2_ [119], TiO_2_ nanotubes [120], and TiO_2_ nanocomposite [121] have been studied as smart drug delivery carriers; also approved in EU as a food additive (E171)	A 20 nm TiO_2_ NPs induced genotoxicity in Syrian hamster embryo fibroblasts at various concentrations ranging from 0.5–10 mg/cm^2^ via the production of ROS as a result of the NPs interaction with the cell membrane [122]. A dose-dependent DNA lesion has been reported upon exposure of HEpG-2 cell to a range of TiO2 NPs concentrations (10–100 *µ*g/mL) [123]. A dose-dependent micronuclei production and DNA strand breakage have been reported in human lymphocytes by comet and micronucleus assay [124].	Genotoxicity of TiO_2_ particles has been reported in mice after 5 days of oral exposure in addition to DNA deletions upon exposure during fetal development [125].

5.	*Iron oxide (Fe*_*2*_*O*_*3*_*) NPs*: magnetite Fe_2_O_3_ NPs is an important candidate for drug delivery and a potential carrier for brain-targeted drug delivery [88]	Induce dose-dependent DNA damage when rat alveolar macrophages and human monocyte cells at concentrations of 5.1 and 10.2 *µ*g/cm^2^ [126].	Micronucleus induction has been demonstrated after administration [127, 128].

6.	*Silica NPs*	Amorphous fumed silica-induced significant oxidative DNA damage in human colon epithelial cells line after 24 h of exposure [129]. Similarly, exposing human lymphoblastoid cells to 100 nm ultrafine crystalline SiO_2_ NPs during 6, 24, and 48 h at a range of concentrations (0-a20 mg/ml) induced genotoxicity [130].	Genotoxicity has been reported in rats after a short period (1 and 3 days) inhalation of a freshly generated aerosolized amorphous SiO_2_ of size 37 and 82 nm, the toxicity was estimated after 24 h to 2 months after exposure [131].

7.	*Organic NPs*: organic nanoparticles could be colloidal, e.g., polymeric NPs, solid lipid NPs, or vesicular, e.g., liposomes	Dendrimers are hyper-branched polymers which have been used as a promising drug delivery vehicle. Cationic dendrimers showed increased oxidative stress and DNA damage in human neural progenitor cells dependent on surface group density and number of particles [132].	

**Table 3:** Neurotoxic effects of some nanoparticles.

Nanoparticles	Toxic effects	References
Carbon nanotubes	Inflammation in the olfactory bulb. Promotes ROS formation, increases oxidative stress, inhibits cell proliferation and apoptosis.	[136, 137]

Silver NPs	Increases oxidative stress and decreases the anti-oxidation capacity of the antioxidative enzymes in frontal cortex and hippocampus of mice.	[138]

Titanium oxide NPs	Induces oxidative stress, neuroinflammation, genotoxicity, neurotransmitters dysregulation, disrupted signaling pathways, and plasticity of the synapse.	[139–141]

Iron oxide NPs	Neuroinflammation, apoptosis, and immune cell infiltration have been reported as a side effect of exposure to iron oxide nanoparticles.	[142–145]

Silica	It causes cognitive dysfunction impairment, synaptic changes, an increase in oxidative stress, and microglial function alteration.	[146, 147]

Organic NPs	It causes dose-dependent inflammation, oxidative stress, neuronal apoptosis, and accumulation in the frontal cortex.	[134, 148–153]

## Data Availability

No data were used to support this study.
